# A Functional *HMOX2* Genetic Variant Is Associated with Resting Diastolic and Mean Arterial Pressure in Healthy Humans

**DOI:** 10.3390/antiox15040518

**Published:** 2026-04-21

**Authors:** Vincent Beauchamps, Julianne Touron, Danielle Gomez-Merino, Adrien Lagraniere, Carine Malle, Marie-Claire Erkel, Damien Léger, Mounir Chennaoui, Fabien Sauvet, Pierre A. Fabries

**Affiliations:** 1 Institut de Recherche Biomédicale des Armées (IRBA), 91220 Brétigny-sur-Orge, France; 2 Vigilance, Fatigue, Sommeil et Santé Publique (VIFASOM), Université Paris Cité, 75004 Paris, France; 3 Académie de Santé des Armées (ACASAN), École du Val-de-Grâce (EVDG), 75004 Paris, France; 4 Hôpital National d’Instruction des Armées Percy, 92140 Clamart, France; 5 Assistance Publique—Hôpitaux de Paris (APHP), Hôtel-Dieu, Centre du Sommeil et de la Vigilance, 75004 Paris, France; 6 US Army Research Institute of Environmental Medicine (USARIEM), 10 General Greene Ave., Natick, MA 01760, USA

**Keywords:** blood pressure, normotensive individuals, sleep, single nucleotide polymorphism, *HMOX2*, heme oxygenase, genetic association

## Abstract

Basal blood pressure (BP) is partly determined by systemic vascular resistance, which is modulated by vasoactive pathways, including gaseous messengers. Carbon monoxide (CO), continuously generated by the constitutive enzyme heme oxygenase-2 (HO-2) encoded by *HMOX2*, promotes vascular smooth muscle relaxation and may contribute to interindividual variability in resting BP. The functional single-nucleotide polymorphism rs4786504_T>C has been associated with higher *HMOX2* expression in C-allele carriers, providing a plausible biological link between genetic variation in the HO-2/CO pathway and vascular redox signaling. We investigated this association in forty young, healthy, normotensive adults studied under controlled laboratory conditions during a 4-day sleep deprivation protocol, with repeated standardized daytime BP measurements (478 observations). Linear mixed-effects models were adjusted for major physiological and behavioral covariates. T-allele carriers (C/T + T/T) exhibited higher diastolic BP (β = +6.08 mmHg, 95%CI [1.32–10.84], *p* = 0.017) and mean arterial pressure (β = +5.28 mmHg, 95%CI [0.28–10.29], *p* = 0.046) than C/C homozygotes, with no effect on systolic BP or heart rate. The association remained consistent across sensitivity and additive genetic models. This hypothesis-generating study provides preliminary evidence in humans, albeit limited by sample size, of a link between a functional *HMOX2* variant and resting BP, consistent with a possible contribution of constitutive HO-2 activity to BP regulation.

## 1. Introduction

It is accepted that family environment, lifestyle, and genetics determine blood pressure (BP) variability [1]. While genome-wide association studies (GWASs) have played a key role in mapping and identifying many common genetic variants associated with specific traits or diseases, the individual contribution of each variant tends to be small [2]. In this context, the study of rare variants and candidate gene approaches targeting well-established physiological pathways may help identify functional genetic variants with a more direct impact on integrative cardiovascular regulation and may contribute to explaining part of the missing heritability of BP [3].

Heme oxygenase (HO) generates the potent vasodilator carbon monoxide (CO), particularly through the constitutively expressed HO-2 isoform in vascular endothelium and smooth muscle cells [4,5,6,7]. In spontaneously hypertensive rats, Kilic-Toprak et al. [8] showed that upregulation of the HO/CO pathway contributes to the systolic BP–lowering effect of exercise, supporting a role for this system in BP regulation. However, in previous studies conducted by Stec et al. [9] on genetically modified mouse models (HO-2 knockout), the contribution of HO-2 to cardiovascular regulation remained controversial. In 2013, the authors suggested that HO-2 might play a sex-specific role in the attenuation of resting BP and target organ damage in renovascular hypertension [10].

The mechanistic vasodilatory effect of CO can be explained by the relaxation of vascular smooth muscle cells (i.e., activation of soluble guanylate cyclase and opening potassium channels, leading to membrane hyperpolarization) [11,12], which increases the diameter of arterioles and reduces systemic vascular resistance (SVR) [6,7,13]. A decrease in SVR is associated with a reduction in arterial BP (BP = cardiac output × SVR) [14]. Given its vasodilatory properties, basal CO production may contribute to interindividual variability in SVR and arterial BP at rest. Genetic variation affecting HO-2 expression and possibly its activity is therefore expected to modulate basal CO levels and influence vascular tone.

The single-nucleotide polymorphism (SNP) rs4786504_*HMOX2*_T>C is located in an intronic region of the *HMOX2* gene, which encodes HO-2 [15]. Carriers of the C allele, which is associated with higher *HMOX2* expression in vitro [15], were shown to display lower hemoglobin concentrations compared with T-allele carriers in hypoxia-adapted Tibetan populations [15], while the ancestral T-allele is associated with lower ventilatory responses (at rest and after exercise) to acute normobaric hypoxia exposure in Europeans [16]. These human genotype–phenotype associations support the functional relevance of this polymorphism across integrative physiological systems [17].

In addition to its vasoactive role, constitutive HO-2 activity contributes to cellular redox homeostasis through continuous endogenous CO production and the generation of biliverdin/bilirubin, which possess antioxidant properties [18,19]. Through these mechanisms, the HO system is increasingly recognized as a central regulator at the interface between vascular function and redox biology [20].

These results suggest a potential involvement of the rs4786504_*HMOX2* polymorphism in the regulatory effects of HO/CO on BP. However, whether genetic variation in *HMOX2* contributes to the physiological regulation of resting BP in normotensive humans has not been investigated. We hypothesized that C/C homozygotes of the rs4786504_*HMOX2* polymorphism would exhibit lower resting diastolic blood pressure (DBP) and mean arterial pressure (MAP) than T-allele carriers. To test this hypothesis, the present study aimed to explore the association between the rs4786504_*HMOX2* polymorphism and arterial BP, independently of known confounding factors. Given the study design of this ancillary study, we were also able to examine whether this association persisted across different sleep conditions (habitual sleep, total sleep deprivation, partial recovery sleep, and total recovery sleep).

## 2. Materials and Methods

### 2.1. Participants

Forty healthy, physically active, normotensive adults were included. Normotension was defined as SBP < 140 mmHg and DBP < 90 mmHg [21,22]. The participants had no active medical condition or clinically significant abnormality. Exclusion criteria included age < 18 or >45 years, BMI > 30 kg/m^2^, alcohol consumption > 1 drink/day more than 5 days/week, and sleep disturbances (Pittsburgh Sleep Quality Index (PSQI) ≥ 5) with habitual time in bed < 6 h. The participants were recruited in a single-center cohort of healthy young adults and were predominantly of European origin according to self-report. Genetic ancestry was not formally assessed.

Written informed consent was distributed to and signed by all participants prior to their inclusion in the study, and the study was approved by the independent *Comité de Protection des Personnes* (CPP) Est III (Nancy) Ethics Committee (IDRCB no. 2023-A01221-44) on 18 August 2023.

#### Sample Size Calculation

This study was part of the larger RECOPS protocol conducted at the French Armed Forces Biomedical Research Institute (IRBA), Brétigny-sur-Orge, France. The primary aim was to examine changes in delta-wave spectral power (1–4 Hz) during a restricted recovery night (3 h time in bed) following sleep deprivation, comparing good and poor performers on a sustained attention task. An a priori sample size calculation indicated that detecting a >10% difference between groups (SD = 9%), with a two-sided α of 0.05 and 80% power, required 13 participants per group (*n* = 26). The present genetic analysis was not part of the original primary outcome, and the study was not powered to detect genotype-related differences in BP nor to test a physiological mechanism.

### 2.2. Study Design and Procedure

BP and heart rate (HR) were measured every 6 h during the daytime under standardized conditions (at 9:00, 15:00 and 21:00) throughout a 4-day laboratory protocol comprising baseline sleep (BS), total sleep deprivation (SD), and two recovery nights (R1 with 3 h of sleep and R2 with 8 h of sleep). Throughout the protocol, the participants were continuously supervised and maintained under tightly controlled environmental conditions, including standardized light exposure, posture, meals, and activity levels.

BP was measured using an automated oscillometric device (Omron M10-IT; HEM-7080IT-E, Omron Healthcare, Kyoto, Japan). This device belongs to the same product family and uses the same oscillometric measurement technology as the Omron 705IT (HEM-759-E), which has been previously validated according to international protocols [23]. According to manufacturer documentation, both devices derive from the same validated measurement platform (https://medaval.ie/resources/EN/devices/Omron-HEM-7080IT.html, accessed on 24 March 2026). Although there are no independent validation studies for the Omron M10-IT, the common features of the two devices may explain its widespread use in numerous clinical and epidemiological studies [24,25]. Measurements were performed following international recommendations for BP measurement [26,27]. BP was assessed on the same arm in all participants, with the cuff adapted to arm circumference and positioned at heart level after a 10 min seated rest in a quiet room, with the back supported and feet flat on the floor. Given the study design, the participants refrained from caffeine, tea, nicotine and physical activity for at least 30 min and were instructed to empty their bladder before each measurement. Three consecutive readings were taken after 1 min of rest between each measurement, and averaged. SBP (mmHg), DBP (mmHg), MAP (MAP = 1/3 SBP + 2/3 DBP; mmHg), pulse pressure (Pp = SBP − DBP; mmHg), and HR (beats per minute (bpm)) were measured and calculated.

### 2.3. Sample Collection and Genotyping

A total of 4 mL of venous blood was collected in K2EDTA tubes (ref. 368861, BD vacutainer) from the antecubital vein and stored at −20 °C until analysis. The rs4786504_*HMOX2* genotype was determined using loop-mediated isothermal amplification with melting curve analysis (LAMP-MC) on a Genie^®^ III instrument and a customized assay kit (LaCAR MDX, Liège, Belgium), as previously described [16]. The participants were classified as C/C homozygotes or T-allele carriers (C/T + T/T) according to a dominant genetic model. This approach was chosen because the very low frequency of the T/T genotype (*n* = 2) precluded reliable genotype-specific comparisons and made codominant or recessive models unstable. The dominant model was therefore considered the most appropriate for the primary analysis, consistent with a prior study investigating this polymorphism [16] and with functional evidence indicating differential *HMOX2* expression between T-allele carriers and non-carriers [15]. An additive model was additionally tested in sensitivity analyses to assess a potential allele-dose effect.

### 2.4. Statistical Analysis

Continuous variables are presented as mean ± SD and categorical variables as counts and percentages. A descriptive analysis of participants’ characteristics was first performed according to the rs4786504_*HMOX2* genotype (C/C homozygotes vs. T-allele carriers). Genotype distributions were tested for Hardy–Weinberg equilibrium. Minor allele frequency and genotype call rate were determined as standard quality-control indicators for the genotyping data. Associations between rs4786504_*HMOX2* and BP were assessed using linear mixed-effects models, including participant as a random intercept to account for within-participant correlation due to repeated measurements. The primary outcomes were DBP and MAP. Fixed effects included genotype (dominant model) and covariates known to influence BP variability, namely sex [28,29,30], age [31], BMI [32,33,34], HR [35], physical activity [22,36,37], sleep condition [38,39,40], and time of day as a proxy for circadian variation [26]. Although smoking status primarily represents a cardiovascular risk factor rather than a direct determinant of resting BP [41,42], it was included as a covariate because of its unequal distribution between genotype groups. A total of 478 BP observations were included in the linear mixed-effects models, reflecting the repeated standardized measurements performed across the 4-day protocol. A minimally adjusted model including age, sex, and BMI was also tested. Additional sensitivity analyses were performed after the exclusion of regular smokers (*n* = 5) from the full model and by testing an additive genetic model (0, 1, or 2 C alleles). To investigate whether rs4786504_*HMOX2* was associated with Pp and HR, we fitted a similar linear mixed-effects model, using Pp and HR as the dependent variables; HR was not included as a covariate in the HR analysis. Collinearity among covariates was assessed using variance inflation factors (VIFs). Model assumptions were evaluated by visual inspection of residual and Q–Q plots. To control for multiplicity, Holm correction was applied to the two primary outcomes (DBP and MAP), whereas secondary and sensitivity analyses were considered exploratory. Analyses were performed using Jamovi software (version 2.5.2.0), and a two-sided *p*-value < 0.05 was considered statistically significant.

## 3. Results

### 3.1. Participant Characteristics by rs4786504_HMOX2 Genotype

Demographic characteristics of the 40 participants are summarized in Table 1. The genotype call rate was 100% and no genotyping errors were detected. Genotype frequencies were C/C: 22 (55%), C/T: 16 (40%), and T/T: 2 (5%), with a minor allele frequency of 0.244. This value was close to that reported for European reference populations. In a gnomAD-derived profile for rs4786504_*HMOX2*, the Non-Finnish European frequency of the common allele is 0.735, corresponding to a minor allele frequency of approximately 0.265 (https://gnomad.broadinstitute.org/, accessed on 24 March 2026). The genotype distribution was consistent with Hardy–Weinberg equilibrium (χ^2^ = 0.18, *p* = 0.67). The two groups differed only in age (*p* = 0.006) and tobacco use (*p* = 0.013).

### 3.2. Association Between rs4786504_HMOX2 and BP Values

#### 3.2.1. Main Analysis

**Figure 1:**
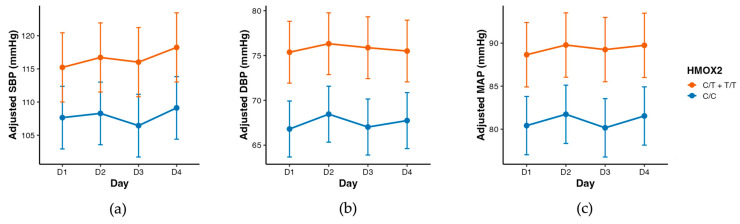
Adjusted systolic (SBP) (**a**), diastolic (DBP) (**b**) and mean arterial pressure (MAP) (**c**) across days (D1: after baseline sleep, D2: after total sleep deprivation, D3: sleep recovery 1 (3 h), D4: after sleep recovery 2 (8 h)) according to the rs4786504_*HMOX2* (HMOX2) single-nucleotide polymorphism. Lines represent estimated marginal means derived from the linear mixed-effects model. Error bars indicate 95% confidence intervals. A significant main effect of rs4786504_*HMOX2* was observed, with no interaction with day. Models were based on 478 blood pressure observations from 40 participants (*n* = 22 C/C homozygotes and *n* = 18 T-allele carriers (C/T + T/T)).

**Figure 2:**
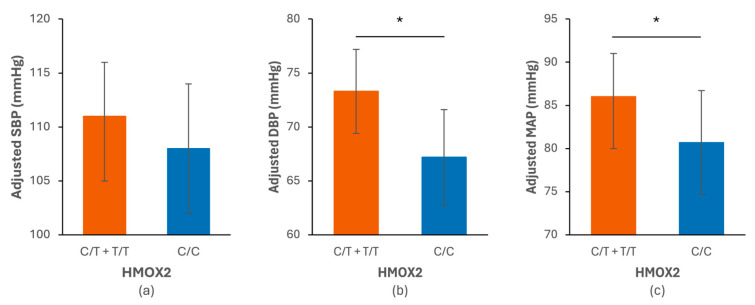
Comparison of adjusted blood pressure values—systolic (SBP) (**a**), diastolic (DBP) (**b**), and mean arterial pressure (MAP) (**c**) according to the genotypes of the rs4786504_*HMOX2* (HMOX2) variant. Estimated marginal means (±95% confidence intervals) of SBP, DBP and MAP according to genotype, derived from the linear mixed-effects model. The values are adjusted to account for repeated measures and covariates included in the models. The models were based on 478 blood pressure measurements in 40 participants (*n* = 22 C/C homozygotes and *n* = 18 T-allele carriers (C/T + T/T)). * *p* < 0.05 for the difference between rs4786504_*HMOX2* genotype groups (C/C homozygotes and T-allele carriers (C/T + T/T)).

Linear mixed-effects models revealed a consistent main effect of the rs4786504_*HMOX2* polymorphism on BP components (Table 2, Figure 1 and Figure 2), with higher DBP (β = +6.08 mmHg, 95% CI [1.32–10.84], *p* = 0.017) and MAP (β = +5.28 mmHg, 95% CI [0.28–10.29], *p* = 0.046) in T-allele carriers compared with C/C homozygotes, independently of sex, age, BMI, smoking status, physical activity, sleep condition, and time of day. Figure 2 presents a direct graphical representation of the genotype effect on adjusted BP parameters based on the estimated marginal means.

After Holm correction for the two primary outcomes, both DBP and MAP remained significant (adjusted *p*_Holm_ = 0.032 and 0.046, respectively).

There was no effect on SBP (β = +3.71 mmHg, 95% CI [−2.61–10.02], *p* = 0.257) in T-allele carriers compared with C/C homozygotes.

No significant interaction with sleep condition or time of day was observed for any BP component (all *p* > 0.521).

Pp was not significantly associated with the rs4786504_*HMOX2* genotype in the fully adjusted model (β = −2.33 mmHg in T-allele carriers compared with C/C homozygotes, 95% CI [−6.20–1.53], *p* = 0.245).

HR was not significantly associated with the rs4786504_*HMOX2* genotype in the fully adjusted model (β = +2.32 bpm in T-allele carriers compared with C/C homozygotes, 95% CI [−3.12–7.76], *p* = 0.408).

No relevant multicollinearity was detected among covariates (VIF < 1.16). Inspection of residual and Q–Q plots did not indicate major violations of model assumptions.

#### 3.2.2. Sensitivity Analysis

Sensitivity analyses using a minimally adjusted model (age, sex, BMI) yielded effect estimates that remained in the same direction and of similar magnitude for DBP (β = +5.77 mmHg, 95% CI [1.04–10.50], *p* = 0.022), and MAP (β = +4.85 mmHg, 95% CI [−0.17–9.88], *p* = 0.066), although the latter did not reach conventional statistical significance for MAP.

The analysis restricted to non-smokers yielded similar effect estimates for DBP (β = +6.35 mmHg, 95% CI [1.86–11.41], *p* = 0.011) and MAP (β = +5.86 mmHg, 95% CI [0.83–10.90], *p* = 0.029).

Under the additive genetic model, each additional C allele was associated with lower DBP (β = −5.38 mmHg per allele, 95% CI [−9.41–−1.34], *p* = 0.013) and MAP (β = −4.68 mmHg per allele, 95% CI [−8.93–−0.43], *p* = 0.038).

In both the minimally adjusted analysis and the analysis restricted to non-smokers, genotype was not significantly associated with SBP (*p* = 0.359 and 0.197, respectively), Pp (*p* = 0.199 and 0.286, respectively) or HR (*p* = 0.560 and 0.387, respectively).

## 4. Discussion

This study provides evidence that the functional *HMOX2* polymorphism rs4786504 is associated with resting DBP and MAP in healthy normotensive adults studied under controlled laboratory conditions during a 4-day sleep deprivation protocol. The association was observed without interaction with sleep condition (baseline sleep, sleep deprivation, sleep recovery), and independently of major physiological covariates. No significant association was observed for SBP, Pp or HR. The absence of genotype × time-of-day (i.e., BP measurement time: 9:00, 15:00 and 21:00) and genotype × sleep-condition interactions indicates that this relationship reflects a stable interindividual difference rather than a state-dependent response.

The variant was selected on the basis of functional data showing altered transcription factor binding and higher *HMOX2* expression in C-allele carriers [15], consistent with its biological plausibility as a determinant of constitutive HO-2 activity. The preferential association with DBP and MAP may be compatible with an influence on vascular tone regulation, rather than on determinants of systolic pressure such as arterial stiffness or stroke volume [31,43], although these physiological variables were not directly measured in the present study. The persistence of the association after adjustment for established determinants of BP—including sex, age, BMI, HR, physical activity, sleep condition, and circadian variation—indicates that the observed association was independent of the measured covariates.

The consistency of the association across the full, minimally adjusted, and additive genetic models supports the robustness of the findings and suggests a coherent allele–dose relationship. The present physiology-driven approach targeting a functional variant for which previous clinical and experimental human data are available strengthens biological plausibility. As smoking status was not balanced between genotype groups and the small number of smokers precluded robust adjustment, this factor could not be fully controlled. Given that smoking can influence vascular tone through oxidative stress–related pathways [44,45], the presence of residual confounding factors or partial mediation cannot be ruled out. Even if this is the case, the consistency of the association observed in the model excluding smokers confirms the consistency of the results.

In this study, the absence of a genotype effect on HR does not suggest major differences in cardiac chronotropic response. MAP is determined by the product of cardiac output and SVR. Under resting conditions in young healthy individuals, cardiac output tends to be relatively stable, and the absence of genotype-related differences in HR argues against major differences in cardiac output. This is consistent with the interpretation that interindividual variation in MAP may more likely reflect differences in vascular tone rather than cardiac function [14], but this assumption cannot be verified in the present study. Therefore, the observed association with DBP and MAP may be compatible with differences in vascular regulation, although no direct measurement of SVR, arterial stiffness, or cardiac output was performed.

Consistently, Pp was not associated with the rs4786504_*HMOX2* genotype. From a hemodynamic perspective, the major direct component of Pp arises from stroke volume interacting with large-artery stiffness and resistance [46,47]. The absence of a genotype effect on Pp therefore does not suggest a major influence of stroke volume, although this was not directly assessed in the present study. The hemodynamic determinants of Pp were not directly assessed and interpretation should remain cautious. Overall, the selective association of rs4786504_*HMOX2* SNP with DBP and MAP, as well as the lack of association with SBP, Pp, and HR, defines a pattern that may be compatible with differences in vascular regulation and a SVR-driven phenotype rather than a phenotype related to cardiac function or large-artery elastic [31,43], but this interpretation remains indirect. On the other hand, this pattern could be consistent with the known vasodilatory action of constitutive HO-2–derived CO on vascular smooth muscle tone in vessels. However, since HO-2 expression, endogenous CO production, and vascular function were not measured, the present results should not be interpreted as demonstrating a causal mechanism. The present results may be consistent with a model in which genetically determined differences in constitutive HO-2 expression might influence basal vascular tone [17]. CO and nitric oxide (NO) share soluble guanylate cyclase as a common molecular target and interact through redox-sensitive mechanisms that regulate NO bioavailability and downstream cyclic guanosine monophosphate signaling in vascular smooth muscle cells [20,48,49]. These mechanisms are described here only to provide biological context.

Experimental models have suggested a role of HO-2–derived CO in cardiovascular regulation, as reduced HO-2 immunostaining in the aorta and increased systolic BP were observed in spontaneously hypertensive rats compared to control rats [8]. However, the role of HO-2 remains debated. Stec et al. showed no significant difference in baseline BP between HO-2 knockout (KO) and wild-type (WT) mice [9], a finding that was subsequently confirmed by the same research group [10]. In this second study, Stout et al. found no difference in BP of sham mice (both male and female) between the two different genotypes, suggesting that HO-2 is dispensable for the regulation of resting BP under basal conditions. However, they showed in the same study that HO-2 may play a sex-specific role in reducing resting BP in HO-2 KO mice that underwent surgery for renovascular hypertension. One possible explanation is the activation of compensatory pathways, including HO-1, which shares overlapping antioxidant and vascular regulatory functions with HO-2 [19,20]. In humans, like HO-2, HO-1 is localized in vessels [50]. A genetic polymorphism in *HMOX1*, the gene encoding HO-1, has been associated with BP levels—particularly DBP—in a normotensive, untreated Chinese Han population [51]. However, in contrast to HO-2, HO-1 is inducible and typically upregulated in response to oxidative stress, inflammation, or tissue injury, and may partially compensate for reduced HO-2 activity [6,19,50,52]. In the present study, participants were healthy and normotensive, and experiments were conducted under controlled conditions without evidence of acute inflammation or stress, making a predominant contribution of inducible HO-1 less likely. Accordingly, while a marked induction of HO-1 appears unlikely, it cannot be excluded, as HO-1 activity was not directly assessed. Therefore, the present findings are consistent with—yet insufficient to establish—a contribution of HO-2-related pathways to the regulation of resting BP in humans.

Given that HO-2 is constitutively expressed and represents a major source of basal endogenous CO [6,17], the observed preferential association with DBP and MAP may be compatible with an influence on SVR, a key determinant of resting arterial pressure [14]; however, this interpretation remains indirect and was not directly assessed in the present study, and the HO-2/CO pathway may therefore be involved in vascular tone regulation [20].

No genotype-related differences in SBP or DBP were observed in our previous study on ventilatory responses to acute hypoxia [16]. In that protocol, BP was not an outcome and was assessed only once at rest on a cycle ergometer using different instrumentation. Moreover, measurements were not standardized according to international guidelines [26], which reduced sensitivity for detecting interindividual differences in basal vascular tone [16].

The present results suggest that interindividual variability in constitutive HO-2 expression [15] may contribute to early physiological differences in vascular tone prior to the onset of overt hypertension (i.e., in normotensive individuals). Although the observed differences in DBP and MAP (approximately 5–6 mmHg) are statistically significant, their magnitude is modest and unlikely to result in clinically meaningful effects at the individual level in a normotensive population. Consequently, in this specific context, these results should not be interpreted as indicating a clinically relevant risk factor. More broadly, these observations extend the physiological relevance of the heme oxygenase system to the regulation of resting vascular function and BP in humans, highlighting the potential contribution of endogenous CO signaling to vascular redox regulation.

### Limits and Perspectives

Several limitations should be considered. The modest sample size limits statistical power for between-genotype comparisons. The present work is an ancillary exploratory genetic analysis. Although the repeated-measures design yielded 478 BP observations and improved statistical efficiency, it did not increase the effective sample size for the genetic comparison, which remained limited to 40 participants.

A post hoc power calculation based on the observed difference in DBP (6.08 mmHg), group sizes of 22 C/C homozygotes and 18 T-allele carriers, and an estimated standard deviation of 7.64 mmHg indicated a statistical power of 80% using a one-sided α level of 0.05, corresponding to the conventional threshold generally considered adequate [53]. However, this estimate should be interpreted with caution, as post hoc power calculations based on the magnitude of the observed effect have significant methodological limitations, do not address the constraints associated with the small sample size, and may lead to misinterpretations. Consequently, the present results should be considered preliminary and hypothesis-generating.

The study population consisted of young, healthy, physically active individuals studied under highly controlled laboratory conditions, which strengthens internal validity but limits generalizability to other populations. Participants were predominantly of self-reported European origin; however, genetic ancestry was not formally assessed using ancestry-informative markers. Self-reported ancestry does not exclude residual population stratification, which may represent a source of confounding in genetic association studies. Therefore, ancestry-related bias cannot be excluded.

As noted above, because HO-2 expression and activity, endogenous CO production, vascular resistance, and arterial stiffness were not assessed, these findings should be interpreted as associative only, without direct evidence of an underlying physiological mechanism. Accordingly, the relationship between rs4786504_*HMOX2* and DBP/MAP should be considered preliminary, and alternative explanations cannot be excluded, including linkage disequilibrium with other loci or CO-independent pathways, such as antioxidant effects related to biliverdin/bilirubin metabolism [6,19].

Despite these limitations, the study benefits from repeated standardized measurements obtained under controlled laboratory conditions. Replication in larger, genetically characterized cohorts and studies including direct physiological measurements will be required to confirm these findings and clarify the underlying mechanisms.

## 5. Conclusions

In this cohort of healthy normotensive adults, homozygous C/C carriers of the rs4786504_*HMOX2* polymorphism had lower resting DBP and MAP, independently of major physiological and behavioral covariates. To our knowledge, these results provide preliminary human evidence for an association between a functional *HMOX2* SNP and resting BP regulation and are compatible with a possible role of HO-2 in BP regulation, although this does not demonstrate it. By identifying *HMOX2* as a potential candidate gene of interest for the physiological regulation of resting BP, this study may support the biologically plausible hypothesis that the HO/CO pathway may contribute to interindividual variability in BP. However, given the exploratory nature of this study and the lack of direct physiological measurements, these results should be interpreted with caution, particularly in light of the limited sample size and the highly selected study population. Replication in independent populations, together with integrative mechanistic studies, will be required to confirm these observations, establish causal pathways, and define the role of *HMOX2* within the polygenic architecture of BP regulation.

## Figures and Tables

**Table 1:** Demographic and clinical characteristics according to rs4786504_*HMOX2*.

Variable	All (*n* = 40)	rs4786504_*HMOX2*		*p*-Value
		C/C	C/T + T/T	
Genotype frequencies (*n* (%))		22 (55)	18 (45)	
Male sex (*n*)		9	12	0.105
Age (years)	31.8 ± 7.6	29.0 ± 7.1	35.3 ± 6.7 *	0.006
Height (cm)	172.0 ± 9.6	172.1 ± 9.9	171.4 ± 9.6	0.824
Weight (kg)	73.3 ± 12.9	72.9 ± 12.3	73.9 ± 14.0	0.796
BMI (kg/m^2^)	24.8 ± 3.4	24.5 ± 3.2	24.8 ± 3.6	0.616
Physical activity (h/week)	5.1 ± 3.4	5.4 ± 3.3	4.7 ± 3.6	0.533
Smokers ≥ 5 CPD (*n* (%))	5 (12.5)	0	5 (27.8) *	0.013
HR (bpm)	70.4 ± 8.7	70.8 ± 8.6	69.9 ± 9.1	0.741

BMI, body mass index; CPD, cigarettes per day; HR, heart rate. * *p* < 0.05 is difference between rs4786504_*HMOX2* allele (C/C homozygotes vs. T-allele carriers (C/T + T/T)). Values are mean ± SD or *n* (%), as appropriate.

**Table 2:** Fixed effects from linear mixed-effects models for blood pressure (BP) outcomes. Values are fixed-effect estimates (β) from linear mixed-effects models and are shown as β with 95% confidence intervals. Sleep condition (Sleep) and time of day (Time) effects are reported as omnibus tests. Interaction terms (Sleep × *HMOX2* and Time × *HMOX2*) were included to test whether the effect of genotype varied across sleep condition or time of day. *p*-Values are presented in the following order: systolic blood pressure (SBP), diastolic blood pressure (DBP), and mean arterial pressure (MAP). All models included identical fixed and random effects, with participant included as a random intercept to account for repeated measurements. β coefficients represent adjusted changes in blood pressure (mmHg) per unit increase of the predictor, unless otherwise specified. Omnibus and interaction tests correspond to global tests for multi-level factors and are therefore reported as *p*-values only, whereas regression coefficients and 95% confidence intervals are provided for fixed effects.

Effect	Comparison/Unit	SBP		DBP		MAP	
		β [95% CI]	*p*-Value	β [95% CI]	*p*-Value	β [95% CI]	*p*-Value
**Fixed effects**							
rs4786504_*HMOX2*	C/T + T/T vs. C/C	3.71 [−2.61–10.02]	0.257	6.08 [1.32–10.84]	0.017	5.28 [0.28–10.29]	0.046
Sex	M vs. F	13.52 [7.91–19.14]	<0.001	2.54 [−1.69–6.78]	0.246	6.21 [1.76–10.66]	0.010
Age	per + 1 year	0.51 [0.11–0.92]	0.018	0.38 [0.07–0.69]	0.021	0.42 [0.10–0.75]	0.014
BMI	per + 1 unit	1.13 [0.30–1.96]	0.011	0.77 [0.14–1.39]	0.021	0.89 [0.23–1.54]	0.012
HR	per + 1 bpm	0.21 [0.09–0.36]	<0.001	0.19 [0.10–0.28]	<0.001	0.20 [0.11–0.29]	<0.001
Physical activity	per + 1 h/week	1.08 [0.27–1.89]	0.013	0.49 [−0.13–1.10]	0.128	0.69 [0.04–1.33]	0.043
Regular smokers	Yes vs. No	−5.59 [−14.75–3.57]	0.239	−3.22 [−10.13–3.69]	0.367	−4.00 [−11.26–3.26]	0.287
**Omnibus tests**							
Sleep condition	Overall effect		0.002		0.246		0.021
Time of day	Overall effect		<0.001		<0.001		<0.001
**Interaction terms**							
Sleep × *HMOX2*	Overall interaction		0.521		0.943		0.799
Time × *HMOX2*	Overall interaction		0.713		0.761		0.743

BMI, body mass index; bpm, beats per minute; HR, heart rate. Models based on 478 BP observations (*n* = 40 participants).

## Data Availability

The data presented in this study are available on request from the corresponding author. The data are not publicly available due to institutional and governmental restrictions, as they originate from the French Ministry of Armed Forces and War Veterans and require authorization for access.

## Abbreviations

The following abbreviations are used in this manuscript:

BMI	Body mass index
BP	Blood pressure
BS	Baseline sleep
CO	Carbon monoxide
CPD	Cigarettes per day
DBP	Diastolic blood pressure
GWAS	Genome-wide association study
*HMOX1*	Heme oxygenase-1 gene
*HMOX2*	Heme oxygenase-2 gene
HO	Heme oxygenase
HO-1	Heme oxygenase-1
HO-2	Heme oxygenase-2
HR	Heart rate
IRBA	French Armed Forces Biomedical Research Institute (*Institut de Recherche Biomédicale des Armées*)
LAMP-MC	Loop-mediated isothermal amplification with melting curve analysis
MAP	Mean arterial pressure
NO	Nitric oxide
NOS	Nitric oxide synthase
Pp	Pulse pressure
PSQI	Pittsburgh Sleep Quality Index
R1	Recovery night 1 (3 h time in bed)
R2	Recovery night 2 (8 h time in bed)
SBP	Systolic blood pressure
SD	Sleep deprivation
SNP	Single-nucleotide polymorphism
SVR	Systemic vascular resistance
